# Non-invasive prostate cancer detection by measuring miRNA variants (isomiRs) in urine extracellular vesicles

**DOI:** 10.18632/oncotarget.8124

**Published:** 2016-03-16

**Authors:** Danijela Koppers-Lalic, Michael Hackenberg, Renee de Menezes, Branislav Misovic, Magda Wachalska, Albert Geldof, Nicoletta Zini, Theo de Reijke, Thomas Wurdinger, Andre Vis, Jeroen van Moorselaar, Michiel Pegtel, Irene Bijnsdorp

**Affiliations:** ^1^ Department of Pathology, VU University Medical Center, Amsterdam, The Netherlands; ^2^ Department of Neurosurgery, Neuro-Oncology Research Group, VU University Medical Center, Amsterdam, The Netherlands; ^3^ Exbiome B.V., 1016 PL Amsterdam, The Netherlands; ^4^ Department of Genetics, Computational Genomics and Bioinformatics Group, University of Granada, Granada Spain; ^5^ Department of Epidemiology and Biostatistics, VU University Medical Center, Amsterdam, The Netherlands; ^6^ Department of Human Genetics, Leiden University Medical Center, Leiden, The Netherlands; ^7^ Department of Urology, VU University Medical Center, Amsterdam, The Netherlands; ^8^ CNR-National Research Council of Italy, IGM, Bologna, Italy; ^9^ SC Laboratory of Musculoskeletal Cell Biology, IOR-IRCCS, Bologna, Italy; ^10^ Department of Urology, Academic Medical Centre, Amsterdam, The Netherlands; ^11^ Department of Neurology, Massachusetts General Hospital and Harvard Medical School, Charlestown, MA, USA

**Keywords:** liquid biopsy, isomiRs, miRNA, RNA sequencing, extracellular vesicles

## Abstract

In many cancer types, the expression and function of ∼22 nucleotide-long microRNAs (miRNA) is deregulated. Mature miRNAs can be stably detected in extracellular vesicles (EVs) in biofluids, therefore they are considered to have great potential as biomarkers. In the present study, we investigated whether miRNAs have a distinct expression pattern in urine-EVs of prostate cancer (PCa) patients compared to control males. By next generation sequencing, we determined the miRNA expression in a discovery cohort of 4 control men and 9 PCa patients. miRNAs were validated by using a stemloop RT-PCR in an independent cohort of 74 patients (26 control and 48 PCa-patients). Whereas standard mapping protocols identified > 10 PCa associated miRNAs in urinary EVs, miR-21, miR-375 and miR-204 failed to robustly discriminate for disease in a validation study with RT-PCR-detection of mature miRNA sequences. In contrast, we observed that miRNA isoforms (isomiRs) with 3′ end modifications were highly discriminatory between samples from control men and PCa patients. Highly differentially expressed isomiRs of miR-21, miR-204 and miR-375 were subsequently validated in an independent group of 74 patients. Receiver-operating characteristic analysis was performed to evaluate the diagnostic performance of three isomiRs, resulting in a 72.9% sensitivity with a high (88%) specificity and an area under the curve (AUC) of 0.866. In comparison, prostate specific antigen had an AUC of 0.707 and measuring the mature form of these miRNAs yielded a lower 70.8% sensitivity and 72% specificity (AUC 0.766). We propose that isomiRs may carry discriminatory information which is useful to generate stronger biomarkers.

## INTRODUCTION

Prostate cancer is the most common form of human cancer, with the second cancer related cause of death in Western males [1]. Human prostate cancer has a complex etiology and despite improved knowledge in the molecular underpinnings of this disease, accurate diagnosis and targeted treatment remains challenging. Suspicion of PCa is generally raised when patients have elevated serum prostate-specific antigen (PSA) levels, and/or abnormal digital rectal examination (DRE) [2]. Subsequently, invasive prostate tissue biopsies are required to determine the histological presence of prostate cancer. While elevated serum PSA levels above 4.0 ng/ml increases the risk of PCa significantly [3], increased PSA levels is not specific for PCa. As a consequence, about 70–80% of the prostate-tissue biopsies are unnecessary [4, 5]. Due to the multifocal growth of PCa within the prostate, there is a chance that PCa is not detected even when multiple prostate-tissue biopsies are taken and accurately examined. It has been estimated that about 20% of PCa cases are not detected with an initial set of biopsies, leading to frequent and often unnecessary repeat-biopsies in patients that have no clinical disease [6]. Apart from the highly invasive nature of taking tumor biopsies, patients may develop infection (sepsis) as a consequence of the procedure [7]. This is becoming a concern regarding the increased antimicrobial resistance, despite the use of pre-biopsy antimicrobial prophylaxis [7]. Therefore, minimally-invasive alternatives for accurate detection of PCa are needed.

Recent studies indicated that extracellular vesicles (EVs), which are small membrane vesicles, are released by (prostate) cancer cells into the extracellular environment. Because of the anatomical location, prostate EVs can be found in urine [8, 9] and their levels can be increased after DRE [10]. Because urine can easily be collected after DRE, analysis of urinary-EV content seems a promising approach for diagnostic testing on PCa as it has several advantages, notably their enrichment for miRNAs that can serve as PCa markers. Apart from prostate cancer, in many cancer types the expression of a prominent class of small gene regulators known as microRNAs (miRNAs) is consistently deregulated. The importance of miRNAs in PCa development is underscored by multiple studies that demonstrated the aberrant miRNA expression in PCa tissues compared to normal tissues [11–13]. Furthermore, miRNAs play important and unique roles with respect to cancer development and progression [14, 15]. EV-associated miRNAs can easily be extracted and quantified by qRT-PCR because they are protected from enzymatic degradation. miRNAs in association with EVs have been described to have diagnostic potential for (prostate) cancer patients. For example, miR-141 and miR-375 are increased in serum of metastatic prostate cancer patients [16–18].

Mature miRNAs are consistently annotated in the public registry miRBase as ∼22 nucleotide-long sequences. Developments in next-generation miRNA sequencing analysis have revealed that in reality many miRNAs in biological samples exist as multiple length variants [19]. Such length variations are usually located at the 3′end of the miRNA sequences [19]. miRNAs with terminal end variations are called isomiRs. IsomiRs comprise many modifications, including elongations, trimmings, sequence variants for example by editing and non-templated additions (NTAs) [12]. IsomiRs are found to varying degrees in deep sequencing analyses depending on tissue origin and disease state [20]. Inexact post-transcriptional processing of miRNAs is thought to be the result of inaccurate Drosha and Dicer processing while isomiRs broaden the complexity in gene-regulatory networks. Even though the exact function of 3′ end modifications are still under investigation, increasing evidence suggests that a proportion of isomiRs is related to disease state possibly due to differences in stability and turnover [21–23]. Most recently, Telonis et al. studied miRNA datasets from TCGA and demonstrated that isomiRs in breast-cancer tissues can separate subtypes [23]. We previously demonstrated that certain miRNA modifications favor or disfavor their release via urinary EVs [24], a rule that may also apply to EVs from different sources as shown by others [25]. However, current knowledge of isomiRs in many biological settings is still nascent and as far as we know a potential association with (prostate) cancer has not been demonstrated.

In the present study, we investigated whether mature miRNAs and their isomiRs have a distinct expression pattern in urine-EVs of PCa patients compared to control men, and analyzed their potential use as biomarker. Here we applied a next-generation sequencing approach to analyze mature miRNAs and isomiR patterns using a recently developed comprehensive bioinformatics pipeline that classifies EV-associated miRNAs including isomiRs [24]. In this study, we addressed the question whether detection of urine-EV isomiRs can reliably report the presence of prostate cancer. Strikingly, our observations show that measuring a small panel of EV-associated isomiRs provides superior clinical information over existing serum PSA measurements and standard detection of miRNAs in urine EVs.

## RESULTS

### Urine EV isolation and miRNA sequencing

**Figure 1:**
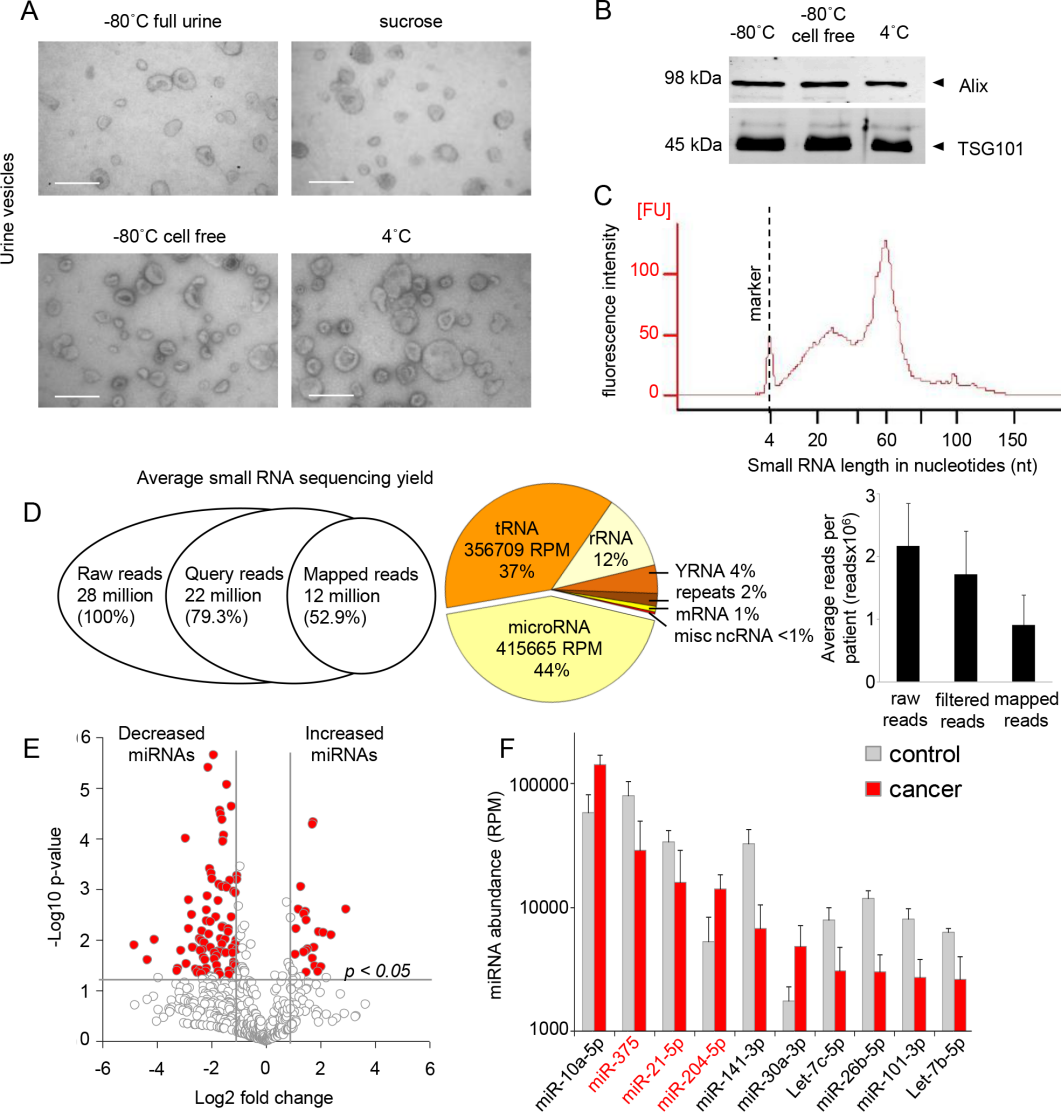
Urine-EV RNA sequencing (**A**) EM pictures of EVs isolated by ultra-centrifugation. Urine stored as full urine, centrifuged over sucrose gradient, after storage as cell free fraction and storage at 4°C. Scale bar = 100 nm. (**B**) Western blot showing vesicle markers Alix and TSG101 in isolated EV-fractions. (**C**) Representative urine-EV small RNA bioanalyzer profile selected for smallRNA sequencing. (**D**) Deep sequencing results overview including the number of reads, mapped sequencing reads and distribution of mapped reads between different ncRNA classes. miRNA (415665 RPM), tRNA fragments (356709 RPM), rRNA fragments (110889 RPM), Y-RNA fragments (41347 RPM), mRNA fragments (8253 RPM), repeat fragments (18632 RPM), and fragments of miscellaneous non-coding RNAs (3028 RPM). (**E**) Volcano plot showing differences between urine-EV miRNAs of control men (*n* = 4) and cancer (*n* = 9) patients miRNAs were classified according to the fold changes (log2 FC), between control men and cancer patients. Vertical dotted lines: miRNA with > 2 fold enrichment in control men or cancer patient urine EVs. (**F**) Top 15 highly expressed miRNAs expressed > 2 fold and *p* < 0.02 between control and cancer patients. miRNAs selected for further analysis are indicated in red.

EVs were isolated by ultracentrifugation and electron microscopy pictures show urinary-extracellular vesicle population with the expected size of 50–150 nm (Figure 1A). A slight improvement was found on the level of background debris when EVs were isolated from a sucrose gradient (Figure 1A). The effect of storage at 4°C, −80°C or −80°C cell free urine on the EV-yield and structure was limited as determined by electron microscopy and by measuring the levels of vesicle markers Alix and TSG101 (Figure 1B). To test for the integrity of isolated EVs and to remove any contaminating RNA associated with cellular debris that may be present in EV pellet we incubated each sample with RNAse-A for 1 hour at 37C. We found that RNAse-A treatment had no effect on small RNA encapsulated in EVs as assessed by the Bioanalyzer small RNA chip (Supplementary Figure S1A), thus confirming the integrity of isolated EVs. However, at this point we cannot formally distinguish whether a slight change observed in small RNA landscape may reflect the RNAse-A activity on RNA molecules ‘stuck’ to the outer membrane of EVs or whether a small amount of EVs have a damaged membrane integrity and therefore renders EV-RNA accessible to RNAse-A activity. We employed Illumina small RNA sequencing (PE100) as a method to examine the total miRNA profile in urine-EVs from 13 patients, including control men, patients with localized PCa and patients with high-risk PCa (the patient characteristics are summarized in Table 1). From these patient samples, we used total RNA isolated (e.g. the highest concentrations of RNA possible per sample) for RNA for library preparation (Figure 1C). A total of 28 million raw reads were obtained (Figure 1D). Of these reads, about 50% could be mapped to the human genome with high confidence (Figure 1D) and with limited variation between the different samples. The reads were mapped to 32,000 individual loci of the human genome (hg19). The length distribution of the mapped miRNA sequences varied from 18 to 25 nt, of which most miRNAs were of 22 nt in length. The most abundant miRNA in urinary EVs of all patients was miR-10b-5p (Supplementary Table S1). miR-10b is highly expressed in various tissues, including kidney and has previously also been observed by others in urine EVs, and also in EVs secreted by the PC-3 prostate cancer cell line [25, 26]. The top 10 of highly expressed miRNAs was comparable between all patient groups (Supplementary Table S1). For all tested urine-EV samples, in addition to miRNAs, which accounted for 44% of the aligned reads, we could classify all other groups of small RNAs. An average of 37% of the aligned reads mapped to the class of tRNAs, and 12% to rRNAs (Figure 1D). Small RNA sequences that mapped to the other ncRNAs such as snRNAs, snoRNAs, piRNA and repeats were represented by less than 2% of the total mapped reads (Figure 1D).

**Table 1:** Clinical characteristics of patients in both cohorts

	Discovery cohort (miRNA sequencing)	Validation cohort (qRT-PCR)
Total number	13	74
Age*, median (range)	65 (54–87)	66 (49–85)
Gleason score**		
6	3	16
7	3	18
8–10	3	14
No cancer	4	26
Clinical T-staging*		
T1	3	16
T2	4	18
T3	2	14
PSA*, median (range)	9.5 (3.9–703)	8.5 (1–424)

* Age (year), T-staging and PSA levels (ng/ml) at time of diagnosis.

** Gleason score after radical prostatectomy tissue or biopsy tissue (when no prostatectomy has been performed).

### Candidate miRNA selection and RT-PCR validation

**Figure 2:**
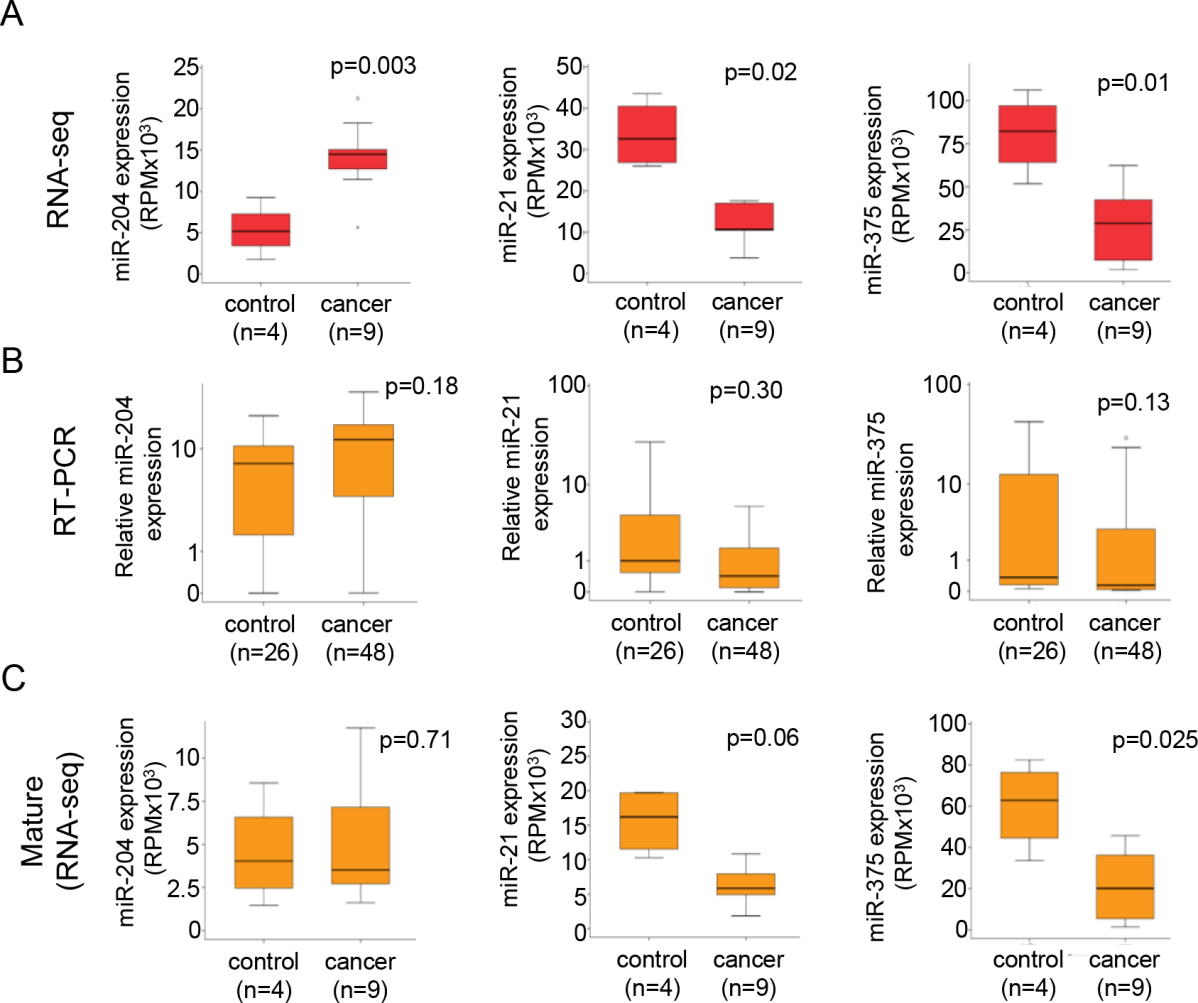
Detection of mature miRNAs is unreliable for validation of RNA sequencing data (**A**) Boxplot of the RNA-sequencing expression (RPM) of the three selected miRNAs, miR-204, miR-21 and miR-375. (**B**) Boxplot showing failure to validate (RT-PCR) the three miRNAs. The data was normalized by ΔCt analysis to control-reference sample, after which the values were transformed to natural-log. (**C**) Boxplot showing the expression of the mature miRNA sequence that was detected by RT-PCR, showing a more similar result as obtained for the RT-PCR detection of the mature-miRNA.

We observed over 200 common miRNAs in urine-EVs of all 13 patients analyzed. Surprisingly, the miRNA abundance in urine-EVs of patients (*n* = 9 patients) with confirmed PCa was lower compared to their relative expression in non-cancer control (*n* = 4 control men) (Figure 1E). For accurate detection of the candidate miRNAs in urine-EVs, high abundance is important to increase the sensitivity. In the 10 most frequent miRNAs that were differentially expressed (fold changed > = 2, (*p* < 0.02)) (Figure 1F), several miRNAs meeting these criteria were previously related to PCa [15]. To further verify the RNAseq expression data with an independent method, we examined the expression levels of 3 candidate miRNAs by sequence specific stem-loop based RT-PCR assay. We selected miRNAs miR-204, miR-375 and miR-21, which had the highest expression in urine-EVs of patients with PCa (Figure 1F and 2A) and were previously related to PCa development and progression [15]. The relative abundance of these three candidate miRNAs was determined using total RNA isolated from urine EVs of 74 patients by RT-PCR. Whereas the results of RNAseq analysis revealed that three miRNA candidates are significantly differentially expressed between two patient groups (Figure 2A), none of those were differentially present between control and PCa patients in qRT-PCR assay analysis (Figure 2B). Because the primers for qRT-PCR were specifically directed towards the mature miRNA sequence (e.g. the miRBase annotated sequence), we analyzed the expression of the mature sequence in our RNAseq data. In agreement with the qRT-PCR data, the reads corresponding to mature sequence of miR-204, miR-21 and miR-375 alone were less differentially expressed in PCa patients compared to control men (Figure 2C). The most striking observation was for miR-204, of which the mature sequence abundance was virtually the same between control and PCa patients and in full agreement with the qRT-PCR results (Figure 2B–2C).

### miRNA-length variants as novel biomarkers

**Figure 3:**
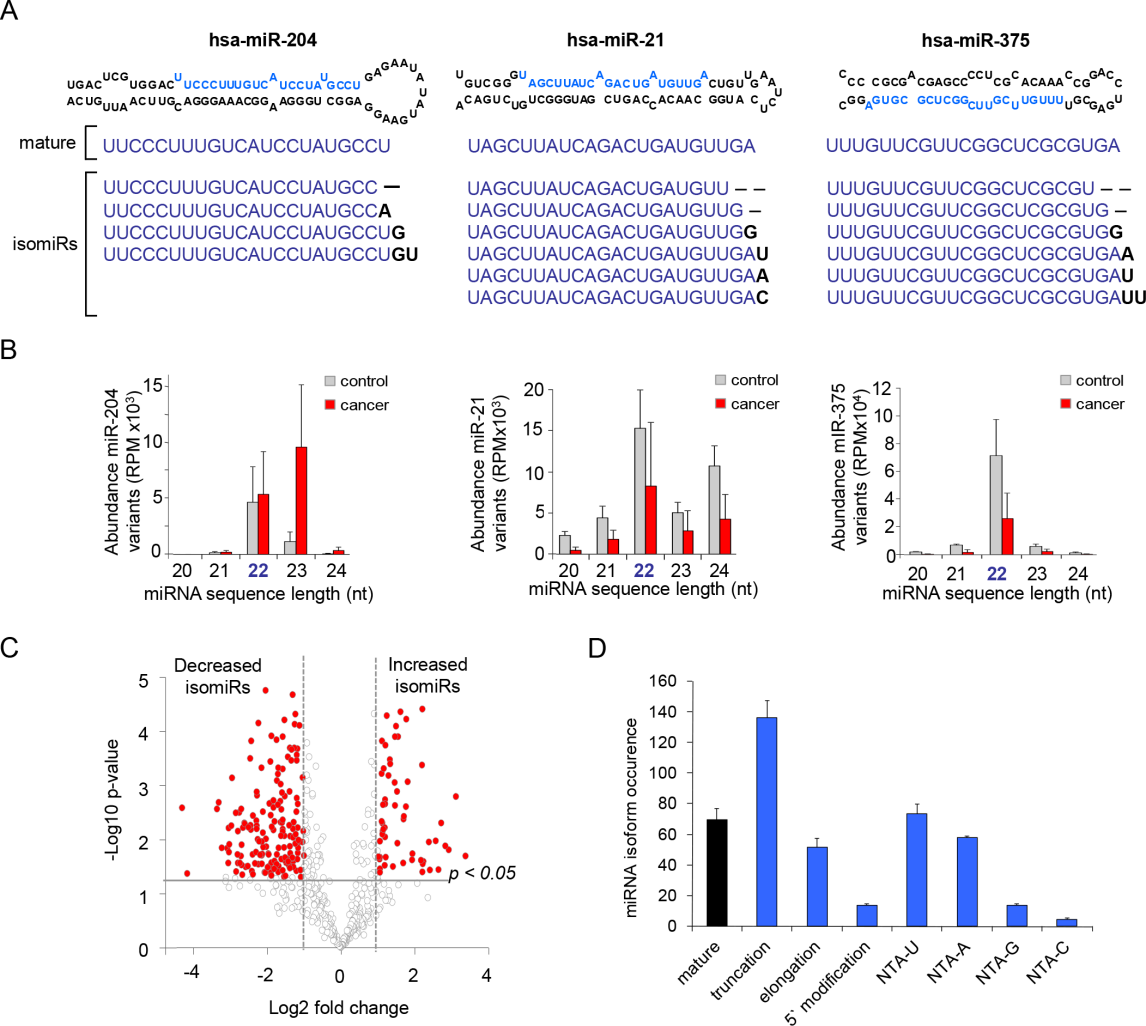
miRNA repertoire consists of multiple length variants (**A**) Examples of miRNA stem loop strucewtures with mature miRNA location in blue. Small RNAseq detected sequences of post-transcriptionally processed isomiRs belonging to miR-204, miR-21 and miR-375. Bold nucleotides represent NTA. (**B**) Relative size distribution of the isomiRs of miR-204, miR-21 and miR-375 showing a clear change in expression of length-variants. (**C**) Volcano plot showing larger differences between urine-EV isomiRs of control men and cancer patients compared to the summed-miRNAs. isomiRs were classified according to the fold changes (log2 FC), between control men and cancer patients. Vertical dotted lines: isomiR with > 2 fold enrichment in control men or cancer patient urine EVs. (**D**) Compared to control men, EVs derived from PCa-patients show differences in the occurrence of miRNA-processing. The isoform-occurrence was calculated by summing the significantly changed isomiR types (e.g. mature, truncation, etc). Most notablenumber of changed variants that were related to PCa were truncation, elongation and non-templated nucleotide additions (e.g. NTA-A and NTA-U).

The presence of isomiRs was a common observation within all urine-EV specimens (Supplementary Table S2). The number of isomiRs was generally increased for miRNAs that were more abundantly present in the urine EVs (Supplementary Figure S2A). miR-204, miR-21 and miR-375 have multiple isomiRs with different lengths (Figure 3A). The miRNA-read-length of miR-204, miR-21 and mir-375 showed clear differences when comparing controls with PCa patient samples (Figure 3B). Importantly, for miR-204 the read length sequences of 23 nt in length were in general the most abundant in PCa patients, while the 22 nt read-length sequences were the most abundant sequences in samples from control men (Figure 3B). Furthermore, in PCa patients, a general decrease of all isomiRs was observed for miR-21 and miR-375.

**Figure 4:**
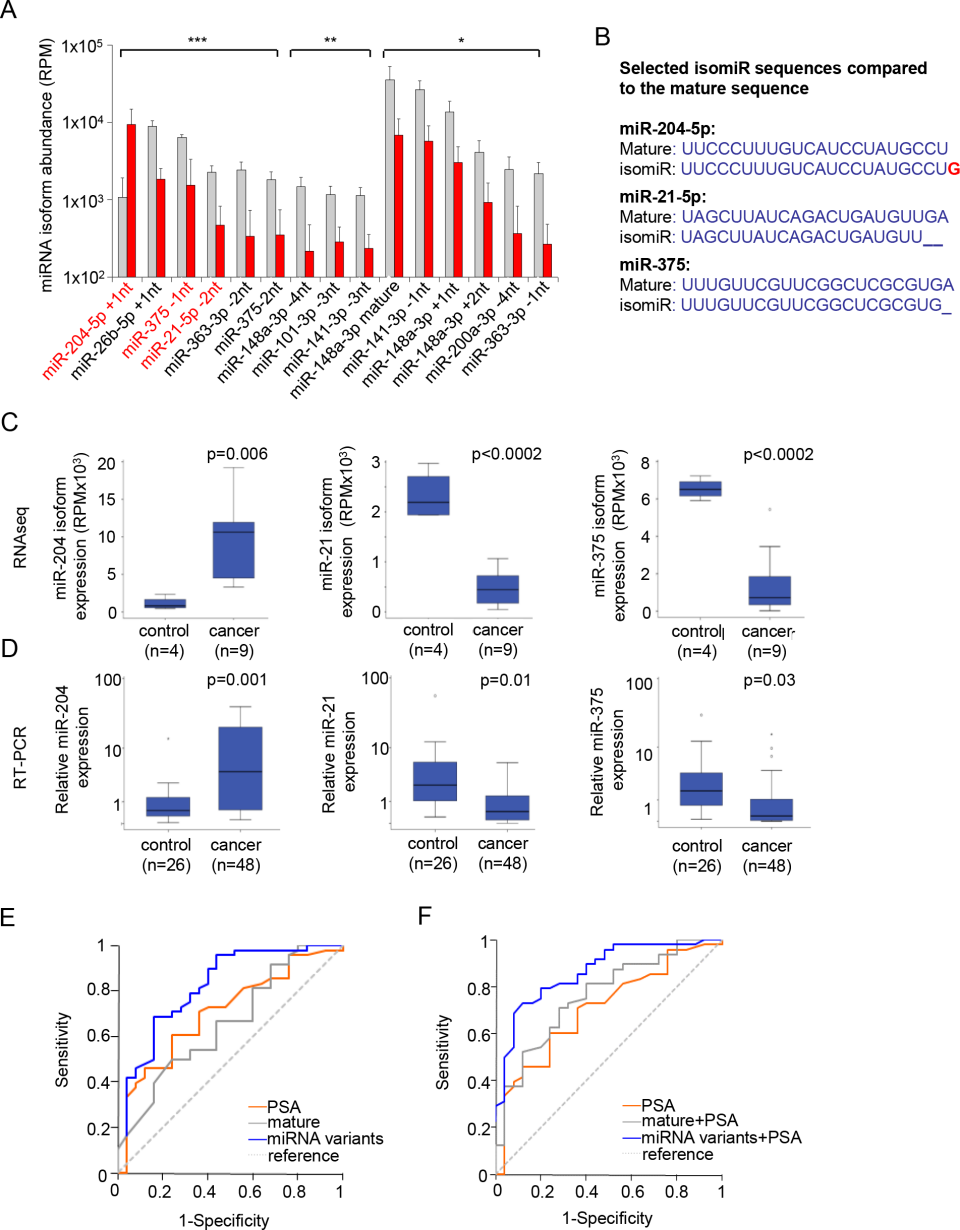
isomiRs improve the specificity for detecting PCa (**A**) Expression of the top 15 isoforms. Isoforms of miR-204, miR-21, and miR-375 were highly abundant and significant. *p < 0.05, **p < 0.01, ***p < 0.001. (**B**) mature and isomiR sequences of miR-204, miR-375 and miR-21 that were selected. (**C**) Boxplot showing the abundance of the isomiRs belonging to miR-204, miR-21 and miR-375 by RNA sequencing. (**D**) RT-PCR validation of the expression of the isomiRs, showing significant differences between control men and cancer patients. (**E**) ROC curves for the combination of the urine-EV isomiRs (blue line), compared to the mature sequence (grey line) and PSA, (orange line). (**F**) ROC curves for the combination of PSA with 3 urine-EV isomiRs (blue line), compared to the mature sequence (grey line) and PSA, (orange line).

Comparative abundance analysis of all miRNA-isoforms suggested that many isomiRs are differentially expressed in patients with PCa compared to control men. Similar as to the summed-miRNA reads (Figure 1E), most of the miRNA-isoforms were decreased (Figure 3C). Of the significantly changed miRNA-isoforms, the occurrence of 3′-end truncation was the most discriminant variant (Figure 3D, Supplementary Figure S2B). Furthermore, the mature miRNA was also commonly differentially expressed between control and PCa patients. With the exception of miR-204, the isoforms with a fold change higher than 4 (*p* < 0.005) and with high relative abundance (> 1000 RPM) were decreased in PCa patients. (Figure 4A). Furthermore, specific miR-375 and miR-21 isomiRs were two of the most significant candidates (Figure 4A).

### isomiR selection and validation

For further validation whether isomiR-specific analysis could improve minimally-invasive identification of PCa, we selected the most discriminant isomiRs of miR-204-5p, miR-21-5p, and miR-375 (Figure 4A–4C). These three isomiRs (Figure 4B) were measured by making use of custom designed stem-loop qRT-PCR primers that are optimized to recognize these isomiR-sequences. We used the same patient cohort in which the mature miRNA sequences were measured by qRT-PCR (Table 1). In agreement with the RNA sequencing data, we now measured a significant difference between control and PCa patients in abundance of the isoforms of miR-204, miR-21 and miR-375 (Figure 4C–4D).

We evaluated the performance of the three miRNA-isoforms by receiver operating characteristic (ROC) analysis and compared the outcome with that of ROCs calculated based on the measurement of mature miRNAs alone. For PSA the predictive accuracy (AUC) was 0.707, which is highly similar to what has been determined before in large patient cohorts [27] (Figure 4E). The predictive accuracy for prostate cancer was decidedly higher for the combined set of isomiRs (AUC 0.821), compared to that of the mature-miRNA panel alone (AUC 0.661) (Figure 4E). The predictive accuracy could be improved further when combining with sPSA to an AUC of 0.866 and 0.766, for the isomiR-set and mature miRNA-set, respectively (Figure 4F). For PSA alone, a concentration of 7.5 ng/ml was the cutoff point that maximized the sensitivity (70.8%) and specificity (60.0%) for the detection of PCa. However, when considering 4 ng/ml as cutoff point, the sensitivity for the detection for PCa was 85.4% and specificity only 32.0%. When decreasing PSA cutoff to 3 ng/ml, this was 95.8% and 24.0%, respectively. On the basis of the predictor model for the sets of 3 isomiRs, a probability of 0.77 was the cutoff point that maximized the sensitivity (72.9%) and specificity (88%) for the detection of PCa in urine-EVs. At this cutoff point, the positive predictive value (PPV) was 92.1% and the negative predictive value (NPV) was 62.9%. For the combined miRNAs at the optimized cut-off point of 0.68 resulted in a lower sensitivity (70.8%) and specificity (72.0%) for the detection of PCa in urine-EVs, compared to that of the isomiR-set. At this cutoff point, PPV was 54.8% and the NPV was 81.0%, which is much lower than the isomiR-panel, showing the strength the selected isomiRs in detection of PCa.

## DISCUSSION

In the present study we demonstrate that urine EVs can be used for minimally invasive tests to detect PCa in suspect individuals. We show that isomiRs present in urinary EVs, more so than mature miRNA sequences are able to detect prostate cancer in suspected patients. Specifically, we identified isomiRs of miR-21, miR-375 and miR-204 that distinguish controls from PCa patients. Using receiver operating characteristic (ROC) analysis we calculated an AUC of 0.821 based on these three isomiRs combined with PSA, showing the potential of these isomiRs for minimally-invasive testing on PCa.

The discovery that miRNAs are sorted into EVs, both by healthy and by cancer cells, has become a general observation made in many laboratories [28, 29]. The applicability of EV-associated nucleic acids, including miRNAs for diagnostic purposes is gaining increasing interest from both clinicians and pharmaceutical industry that seek minimally-invasive diagnostics [30]. A large variety of biofluids such as blood, seminal fluid, breast milk, sputum, cerebrospinal fluid and also urine are known to contain (secreted) miRNAs. Urine is an attractive biofluid as its collection is non-invasive and contains a high concentration of EVs produced by a selected group of organs (prostate, bladder, kidneys and urothelial cells). This is in contrast with blood serum and plasma as biofluids that contain EVs from all organs of the body and are dominated by platelet-derived vesicles [31]. However, in some patients the EVs yield was extremely low, even though all patients had received DRE. This rate is comparable to observations of others [10, 27, 32, 33]. Counter intuitively this may, in part, be related to higher amount of liquid consumption. This has previously been described in studies that analyzed urine [27] and sediments [34]. Fluid restriction may lead to an increase in EV yield. Furthermore, it has been suggested that pre-amplification procedures prior to RT-PCR may increase the cDNA number and thereby improve the detection [33].

With a genome-wide small RNA (Illumina) sequencing analysis, we were able to identify numerous small RNA species present in urine EVs. Apart from the identification of well annotated miRNA-related sequences, the other dominant small RNA sequences mapped to human genome appear to be short fragments of known RNA classes such as tRNA, rRNA, Y RNA and mRNA. The presence of tRNA fragments in urine and sera of cancer patients has been previously documented [35] and seems to correlate with high turnover of tRNAs in cancer tissue. Although the tRNA fragments are present in high amounts in prostate biopsies from metastatic patients [36] and their generation may be androgen-dependent [37], the molecular basis behind their biogenesis, function and potential use as biomarker in urinary EVs remains to be further elucidated.

During the analysis of small RNA class distribution, we observed that small RNA sequences belonging to miRNA class exhibited the most significant deregulation between samples of control men and PCa patients. Among those we identified miRNA sequence variants commonly referred to as isomiRs [38]. Although the biological relevance of isomiR expression in cancer development and progression is not well understood, we found that isomiRs are present at different relative frequencies in urine EVs of PCa patients compared to urine EV samples from control men. An additional conclusion of this study is that detection of mature miRNA sequences as listed in miRBase using dedicated stemloop RT-PCR does not always correspond with RNAseq results. This seems to depend on the analysis thresholds that are applied and could explain why some miRNAs fail as cancer biomarkers in validation studies [39]. This underlines the importance of analyzing the presence and abundance of certain isomiRs when validation by stemloop RT-PCR is preferred [38]. Even though the stem-loop PCR primer method is in theory useful for detecting defined 3′-end isomiR-sequences, cross-detection of related miRNA sequences may occur [38]. Importantly, the isomiRs that we selected in this study as potential PCa biomarkers were selected for their differential abundance in urinary samples obtained from high-throughput sequencing data. We validated the RNA-sequencing data observed differences in abundance for isomiRs and their mature forms using stemloop primers [40]. In general, we measured several-fold differences in abundance between the mature vs isomiRs in concordance with the RNA-sequencing read abundance, suggesting that cross-detection is probably does not have a significant impact on the PCR measurements. A small percentage of cross-detection may still occur, and can therefore not be excluded at this stage. We previously reported that many miRNAs are generally comparably expressed in (tumor) cells and detected in their secreted EVs, while other miRNAs show specific cellular retention or abundance in EVs [24]. More specifically, we found that 3′uridylated miRNAs are more abundant in EVs than could be expected from their relative abundance in producing tumor cells. In contrast, miRNAs that are 3′adenylated are generally underrepresented in EVs compared to their relative expression within the cells [24]. This disruption is emphasized by a recent observations by Boele et al. [41], which described that the poly(A) polymerase PAPD5-mediated adenylation of miRNA-21 is disrupted in cancer. A disruption of post-transcriptional processing of miRNAs can affect their stability, activity, RISC-loading potential, turnover, or affect their enrichment in secreted EVs, and as a consequence measuring of isomiRs may be useful for cancer-specific detection. Notably, the study by Boele and colleagues could explain why several PCa-related miRNAs are actually less abundant in urine EVs even though these are upregulated in cancer tissues. Furthermore, these decreased PCa-related miRNAs in urine were also observed previously by Sapre *et al.* [39]. Additional studies are essential to determine the physiological significance of post-transcriptional miRNA modifications, EV sorting and the role of these processes in cancer development and usefulness for liquid biopsy tests. The modifications such as non-templated nucleotide additions are generated by RNA-modifying enzymes called ribonucleotidyl transferases (rNTAs) but their role in cancer remains largely unexplored.

In conclusion, in this study we demonstrate that detection of EV-associated isomiRs could allow minimally-invasive diagnosis of PCa patients. If validated in larger follow up studies, the isomiRs identified in this study could be useful to predict which patients suspected of PCa require a tissue biopsy and which patients are unlikely to suffer from PCa and may continue minimally-invasive monitoring. Addition of our isomiR panel to existing urinary tests (i.e. PCA3, Quattro, ERG) [27, 42] or those in development, may improve the detection accuracy for PCa.

## MATERIALS AND METHODS

### Urine and extracellular vesicle collection and isolation procedures and transmission electron microscopy (TEM)

For optimization experiments, urine was collected from healthy individuals and stored at 4°C (7 days), at −80°C (7 days) or alternatively centrifuged (500 × g and 2000 × g) at 4°C before −80°C storage. For miRNA expression analysis, we collected 20–90 ml of urine from patients (from July 2012) diagnosed with prostate cancer (Gleason score was adjusted when prostate surgery was performed) or control men (without prostate cancer, confirmed by biopsy). Men who were scheduled for initial or repeat prostate biopsies, based on elevated PSA levels or abnormal DRE were included. Risk-classification was performed according to D'Amico [43]. First catch urine after DRE was collected. Exclusion criteria were previous medical therapy against prostate hyperplasia, chemotherapy, presence of any other cancer type, any previous therapy concerning the prostate (e.g. TURP). Urine was collected after signed informed consent and approval of the medical ethical committee of the VU University Medical Center and stored at −80°C. About 20–90 ml of urine was used for EV isolation, and urine-EVs were isolated by differential (ultra)centrifugation (UC) as described previously [8]. In brief, urine was centrifuged at 20000 × g for 30 min at 4°C to remove the debris. The supernatant was subsequently used for EV isolation by ultracentrifugation at 100000 × g for 90 min at 4°C. The EV pellet was washed in PBS and subsequently centrifuged again at 100000 × g for 90 min at 4°C. When sucrose was used, the EV pellet was dissolved in 30% sucrose and layered on top of 40% sucrose. The exosome layer was extracted from the interfaces, and pelleted at 100000 × g ultracentrifugation for 90 min. Directly after centrifugation, EVs were treated with RNAse-A (4 μg/ml at 37°C for 1 h). Treatment with RNAse-A hardly affected the small RNA profile as determined with Agilent bioanalyzer small RNA chips (Supplementary Figure S1). Ultrastructural evaluation of urine-EVs was performed by transmission electron microscopy (TEM) as described previously [24].

### Protein analyses

Western blot was performed as described previously [8]. The membranes were incubated with mouse anti-Alix (1:500; #2171, Cell Signaling) or goat anti-TSG101 (1:1000; #sc-7964, Santa Cruz Biotechnology, USA).

### Preparation of RNA samples and library construction for deep sequencing

Total RNA was isolated from 13 patients (Table 1) using Trizol LS, according to manufacturer's instruction and small RNA concentration was determined using a small RNA Bioanalyzer Chip (Agilent, Santa Clara, CA, USA). Preparation and sequencing of cDNA libraries was performed using 200–600 ng of small RNA from total RNA samples, according to manufacturer's instruction (Illumina, San Diego, CA, USA) and was performed as described previously [24]. In brief, 13 unique barcode sequences were applied for simultaneous analysis of multiple samples on one lane. The cDNA sequence library yield were measured on an Agilent 2100 Bioanalyzer (Agilent) and the samples were pooled in equimolar concentrations for the sequencing run. Sequencing was performed paired end 100 (PE100) cycles on a HiSeq 2000 (Illumina).

### Data processing deep miRNA sequencing

The expression profiling of miRNAs was performed by means of the sRNA toolbox implementation of sRNAbench, which is the successor of miRanalyzer as described previously [24, 44, 45]. In brief, the processing of the fastq format files included adapter trimming, deletion of reads < 15 nt, and to collapse all reads with identical sequences into one entry (unique reads). The read count assigned to each unique read represents the number of times the corresponding molecule has been sequenced. The reads were aligned using two approaches: all reads alignment to the human genome (hg19 from UCSC), using the bowtie seed alignment allowing one mismatch or all reads annotation by using known RNA databases (to assign reads to different RNA classes). Using one mismatch approach of mapping to the human genome, the seed alignment method avoids that strongly modified molecules (e.g. isomiRs) fail to map, which would impede their detection [45]. Finally, we calculated the RPM (Reads Per Million) expression value for 1. Mature miRNA sequences as sum of all reads that map to a specific miRNA and 2. Individually assigned miRNA sequences (canonical or per isoform type) using the total number of miRNAs mapped reads for normalization. This value is therefore independent of the total read yield of the sample and it is not affected by the relative frequency of other small RNAs.

### Statistical analysis NGS data

To compare abundance for each miRNA between different patient groups, the observed counts were fitted in a generalized linear model using the R package edgeR [46]. The model included not only common and trend dispersion, but also tagwise dispersion estimation, allowing thus for extra variability due to inter-library fluctuation. The *p*-values corresponding to the likelihood-ratio test statistic for the sample type effect were corrected for multiple testing using Benjamini-Hochberg's false discovery rate. We calculated the log2 ratios of expression values between control group and PCa-patient group, and candidate miRNAs were selected based on log2 > = 1, and *p*-value < = 0.05. The miRNA-isoform occurrence was determined by summing the times an isoform of specific type (e.g. trimming, elongation, 5′-modification, non-templated nucleotide additions (NTA) NTA-U, NTA-A, NTA-G, NTA-C and mature sequence) was present in the urine-samples that were significantly changed > = 2 fold difference.

### RT-PCR

Urine EV-RNA (50 ng/sample) from 74 patients (Table 1) was used for reverse transcriptase, which was performed according to manufactures instruction (Life Technologies). Primers used were miRNA assays from Life Technologies # 4427975 for the mature sequences. For Custom primers, Life Technology primers were ordered, miR-21-5p-isomiR (UAGCUUAUCAGACUGAUGUU) #4398987, miR-375-isomiR (UUUGUUCGUUCGGCUCG CGUG) #4398987, miR-204 isomiR (UUCCCUUU GUCAUCCUAUGCCUG) #440886. RT-PCR was performed using 3 μl of diluted cDNA, according to manufacturer's instruction using Light Cycler (Roche). Analysis on expression data of the selected miRNAs by RT-PCR were performed with SPSS version 20.0 and with edgeR [46]. The cDNA of control samples was pooled and used as a reference. The data was normalized by ΔCt analysis to reference, after which the values were transformed to natural-log.

### Statistical analysis

Statistical differences between patient groups was performed in SPSS20.0 and values were considered significantly differentially expressed when *P* < 0.05. Normalized RT-PCR data were used to classify samples according to disease status (healthy vs. cancer) by means of a logistic model. Five different models were considered according to the covariates included: PSA as the only covariate; the set of 3 mature miRNAs; the set of 3 mature miRNAs plus PSA; the set of 3 isomiRs; and the set of 3 isomiRs plus PSA. Each model was used to classify patients using a leave-one-out cross-validation (LOOCV) approach. This involved leaving one patient out of the data at a time, then fitting the model to the patients included, and subsequently computing the predicted probability of disease for the patient that was left out. All predicted probabilities were computed per model, ROC curves were constructed by varying the threshold above which a sample is classified as having the disease between 0.01 and 0.99. For each possible threshold value, sensitivity and specificity are computed. From the ROC curves, areas under the curve (AUC) were determined.

## SUPPLEMENTARY MATERIALS FIGURES AND TABLES


