# Supplementary material for: Non-invasive prostate cancer detection by measuring miRNA variants (isomiRs) in urine extracellular vesicles
Source: Oncotarget. 2016 Mar 16;7(16):22566–78. doi: 10.18632/oncotarget.8124 (PMC5008382; doi:10.18632/oncotarget.8124)
Supplement: Supplementary file 1 [file oncotarget-07-22566-s001.pdf]

Non-invasive prostate cancer detection by measuring miRNA variants (isomiRs) in urine extracellular vesicles

Supplementary Materials

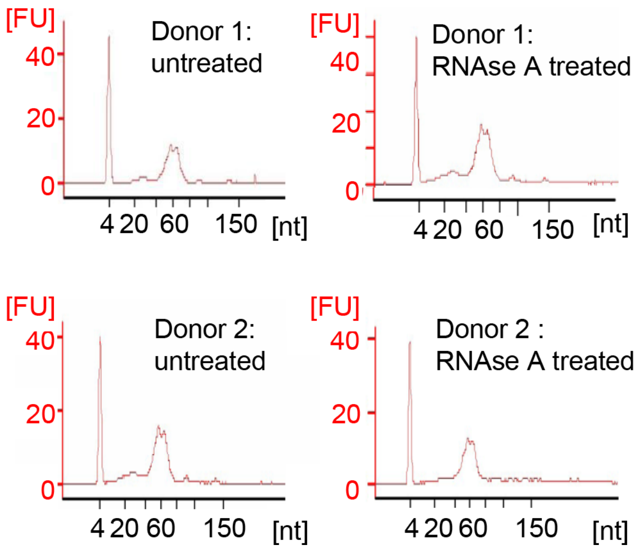

Supplementary Figure S1: Effect of RNAse A treatment on the small RNA profile (bioanalyzer chip) of urinary EVs from 2 different donors, showing a limited change in small RNA levels. This suggests that a small amount of random RNA molecules were attached to the outer surface membrane of the urinary extracellular vesicles.

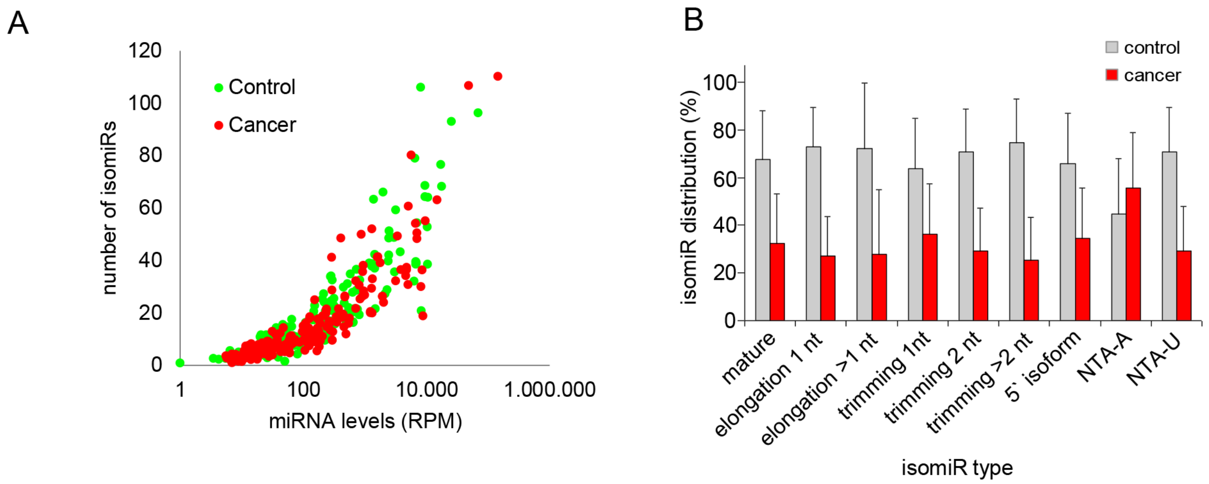

Supplementary Figure S2: (A) Relation between miRNA expression and number of isomiRs. miRNAs that are expressed at higher levels in general have more isomiRs. (**B**) Distribution of isomiR-type in RNA-sequencing data, comparing control to prostate cancer urine-EV profiles.

Supplementary Table S1: Top 10 most abundant miRNAs

miRNA	control	Cancer
miR-10b-5p	259434 ± 49444	427917 ± 56335
miR-10a-5p	65230 ± 25420	178506 ± 18601
miR-375	91711 ± 27977	26157 ± 16257
miR-30a-5p	40426 ± 17671	56868 ± 6408
miR-22-3p	30890 ± 8601	28541 ± 7246
miR-148a-3p	60214 ± 25067	13243 ± 7898
let-7f-2-5p	24312 ± 9371	21974 ± 8800
miR-26a-5p	34124 ± 6021	26002 ± 8328
miR-21-5p	38019 ± 9006	13883 ± 4909
miR-27b-3p	26812 ± 8460	16659 ± 1210

Values in reads per million (RPM) ± standard deviation.

Supplementary Table S2: Top 10 miRNAs with isomiRs

miRNA	control	cancer
miR-10b-5p	96 ± 10	110 ± 26
miR-10a-5p	77 ± 11	107 ± 22
miR-92a-3p	106 ± 24	80 ± 21
miR-30a-5p	64 ± 8	63 ± 26
miR-375	93 ± 10	55 ± 24
miR-27b-3p	54 ± 4	54 ± 12
miR-151a-3p	63 ± 10	52 ± 9
miR-26a-2-5p	69 ± 11	50 ± 12
Let-7b-5p	66 ± 10	50 ± 14
miR-192-5p	42 ± 7	49 ± 26

Values are the average number of isomiRs for each miRNA ± standard deviation.
